# Our Journal

**Published:** 1871

**Authors:** 


### *Our Journal.*

As announced in the last number, arrangements are in progress, not yet completed, but upon a footing which makes it a certainty, by which this Journal will be placed under a management which will secure it a merit that will deserve a large and permanent patronage throughout the South. Among its editors and co-laborers will be found prominent and influential gentlemen, not only throughout this State, but from the various sections of the United States. There has probably never been a period when so favorable an opportunity was offered for the successful establishment of a Medical Journal of the highest order, in the Southern country, and of this opportunity we propose to avail ourselves without regard to the special interest of any institution, organization or individual.

